# Populatieverschillen in de volledigheid en betrouwbaarheid van COVID-19-registratiedata: De invloed van testbeleid, testbereidheid en registratiebeleid vanuit een epidemiologisch perspectief

**DOI:** 10.1007/s12508-022-00358-7

**Published:** 2022-08-03

**Authors:** Babette van Deursen, Ester A. L. de Jonge

**Affiliations:** 1 afdeling Infectieziektebestrijding, GGD Rotterdam-Rijnmond, Rotterdam, Nederland; 2 onderdeel van Dienst Gezondheid & Jeugd ZHZ, cluster Regie, Onderzoek & Advies, GGD Zuid-Holland Zuid, Dordrecht, Nederland

**Keywords:** COVID-19, registratiedata, datakwaliteit, populatieverschillen, datagestuurd beleid, Data quality, Population differences, COVID-19 registration, Data driven policy

## Abstract

De complexe interactie tussen het beleid en de kwaliteit van registratiedata vormde tijdens de COVID-19-pandemie een uitdaging voor GGD-onderzoekers. Beleidskeuzen gericht op populatiespecifieke testlocaties en de selectieve registratieplicht van negatieve testresultaten leidden tot populatieverschillen in datakwaliteit. Populatieverschillen in de besmettingsgraad konden daardoor niet betrouwbaar worden vastgesteld. Dit belemmerde de ontwikkeling van relevante sturingsinformatie voor beleidsmakers in de publieke gezondheidssector.

## Abstract (en)

During the COVID-19 pandemic, the bidirectional relationship between policy and data reliability has been a challenge for researchers of the local municipal health services. Policy decisions on population specific test locations and selective registration of negative test results led to population differences in data quality. This hampered the calculation of reliable population specific infection rates needed to develop proper data driven public health policy.

## De snelle opkomst van datagestuurde besluitvorming

Lokale overheden, zoals gemeenten en hun gemeentelijke gezondheidsdiensten (GGD’en), experimenteren al jaren met een datagestuurde ontwikkeling van beleid en besluitvorming. Tijdens de COVID-19-pandemie werd de prominente rol van data in besluitvorming ook zichtbaar voor het grote publiek. Zo werd dagelijks gerapporteerd over het aantal nieuwe besmettingen, ziekenhuisopnamen en overleden personen. Ook werden complexe begrippen, die eerder vooral weggelegd waren voor wetenschappers, zoals ‘het R‑ getal’, een vast onderdeel van de nationale COVID-19-persconferenties. Het gebruik van termen als ‘routekaarten’ en ‘signaalwaarden’ in deze persconferenties lijken te impliceren dat de gebruikte data een hoge betrouwbaarheid hebben. Een hoge betrouwbaarheid is echter niet altijd vanzelfsprekend. In het publieke debat ligt de focus op de relevantie van data voor besluitvorming en beleid. In dit artikel illustreren wij het omgekeerde, namelijk hoe besluitvorming en beleid ook de relevantie van data kunnen bepalen.

Het probleem van variatie in databetrouwbaarheid werd aanvankelijk vooral gevoeld door epidemiologen en onderzoekers publieke gezondheid van de GGD, omdat zij verantwoordelijk waren voor het aanleveren van betrouwbare sturingsinformatie aan beleidsbepalers. Aan de ene kant was de druk groot om snel betrouwbare sturingsinformatie over bijvoorbeeld testbereidheid of hoogrisicogroepen te bieden. Aan de andere kant hadden onderzoekers geen invloed op de dataverzameling en de betrouwbaarheid van deze data voor het ontwikkelen van sturingsinformatie. Hierdoor ontstond een spanningsveld.

Ter onderbouwing van dit artikel gebruiken we een onderzoek uit de GGD-regio’s Rotterdam-Rijnmond, Zuid-Holland Zuid en Zeeland, gefinancierd binnen de onderzoekslijn ‘Zorg en preventie voor kwetsbare burgers’ van het Corona en COVID-19-programma van ZonMw [1].

## Doel en casusbeschrijving

Het primaire doel van dit artikel is illustreren hoe de interactie tussen test- en registratiebeleid en de navolging daarvan de kwaliteit van COVID-19-registraties heeft beïnvloed. Onder datakwaliteit verstaan we een combinatie van volledigheid van de brondata en betrouwbaarheid en relevantie van de daarmee berekende sturingsinformatie. Op basis van onze bevindingen formuleren we een aantal aanbevelingen waarmee de kwaliteit van registratiedata in een volgende gezondheidscrisis verder kan worden verbeterd.

Het oorspronkelijke onderzoekdoel van onze casus was het vaststellen van populatieverschillen in gediagnosticeerde COVID-19-prevalentie in Zuidwest Nederland, met extra aandacht voor woon- en werkomstandigheden. Om dit doel te bereiken zijn COVID-19-registratiedata van deelnemende GGD’en op persoonsniveau gekoppeld aan microdata over sociaaleconomische positie (SEP), demografie, woon- en werkomstandigheden van het Centraal Bureau voor de Statistiek (CBS) [2]. Onder registratiedata verstaan we alle registraties van GGD-teststraatbezoeken en alle registraties van positieve testuitslagen. Datasets gekoppeld op individueel niveau geven andere informatie dan datasets gekoppeld op een geaggregeerd niveau, zoals wijkniveau. Ze zijn geschikt voor het vaststellen van kenmerken van individuen die samenhangen met een hogere besmettingsgraad. Daarmee wordt voorkomen dat een verband op wijkniveau ten onrechte wordt geëxtrapoleerd naar het individuele niveau (‘ecologische vertekening’) [3]. Dit type vertekening treedt op wanneer inwoners van de ene wijk een hoger risico liepen op een COVID-19-besmetting dan inwoners van een andere wijk, veroorzaakt door andere kenmerken van de wijk dan de onderzochte determinanten. De determinanten die in onze casus zijn onderzocht waren SEP, demografie, en woon- en werkomstandigheden. Het koppelen van data op persoonsniveau wordt mede daarom ook door het CBS gestimuleerd en gefaciliteerd. Resterende vertekening van de resultaten veroorzaakt door kenmerken van individuen die niet werden gemeten, kan echter niet volledig worden uitgesloten.

Dit project werd geleid en uitgevoerd door twee epidemiologen van de GGD en uitgevoerd in samenwerking met collega’s van de afdelingen infectieziektebestrijding, beleid en een academische partner. De onderzoeksperiode liep van juni 2020 tot en met april 2021.

### Heersend registratie- en testbeleid relevant voor onze casus

**Figure 1:**
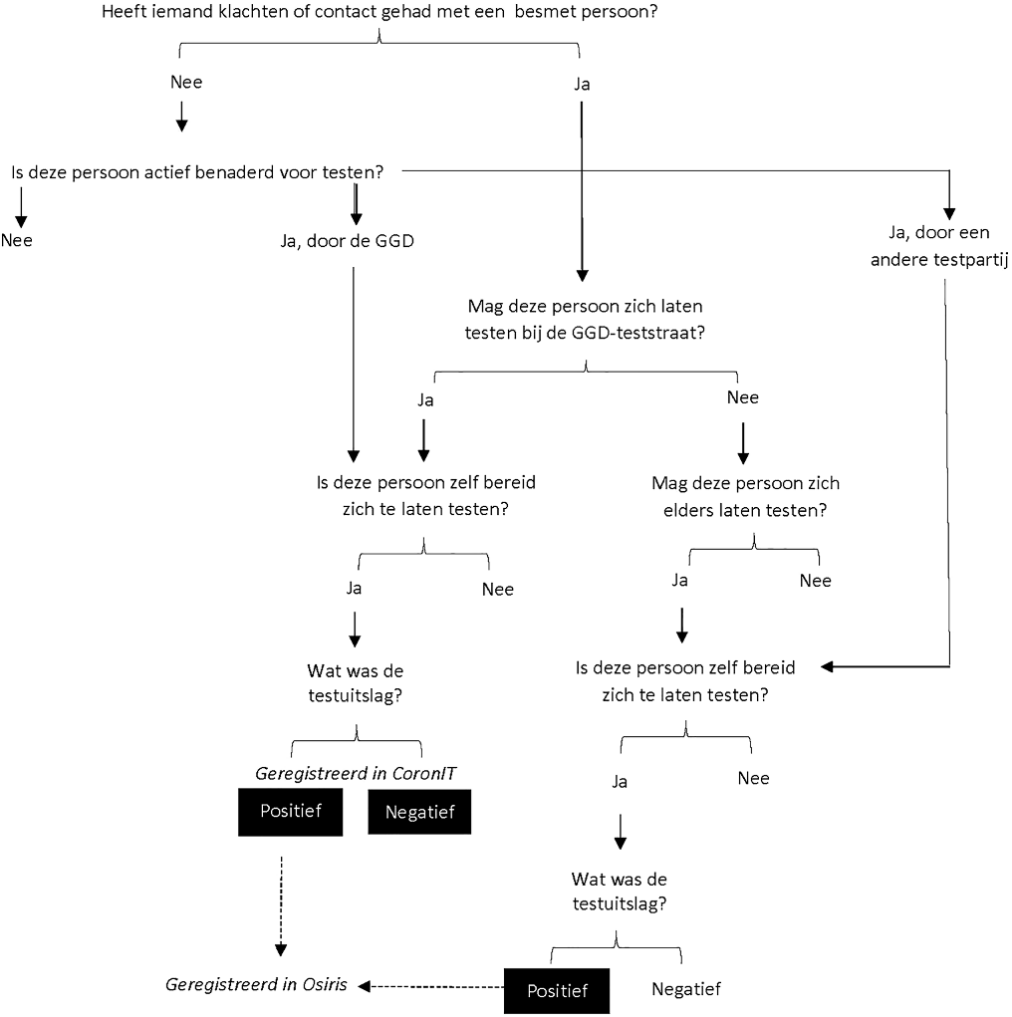

De datakwaliteit van deze registraties werd beïnvloed door twee vormen van beleid. Ten eerste door het registratiebeleid, waarin werd vastgelegd welke uitslagen in de officiële registraties terechtkwamen. Ten tweede door het testbeleid, waarin werd vastgelegd wie in aanmerking kwam voor een test. De mate waarin het beleid in de praktijk werd opgevolgd was hierin een aanvullende complicerende factor (zie de schematische weergave in fig. 1).

Het registratiebeleid was stabiel over de onderzoeksperiode van onze casus. Inwoners die zich lieten testen bij de GGD-teststraat werden, ongeacht de uitslag, geregistreerd in het registratiesysteem CoronIT. Inwoners met een positieve testuitslag werden, ongeacht de testlocatie, geregistreerd in het registratiesysteem Osiris. Daardoor zijn de negatieve testuitslagen voor alle inwoners die buiten de teststraten van de GGD werden vastgesteld, *niet* geregistreerd (selectieve ontbrekende waarden). Andere testlocaties zijn onder meer ziekenhuizen, verzorg- en verpleeghuizen en commerciële teststraten. In de grensregio’s speelde een aanvullend effect van het registratiebeleid. Er was geen meldingsplicht van kracht tussen België en Nederland, waardoor de testuitslag van inwoners die in België getest werden, niet in Nederland geregistreerd werd.

Het testbeleid was veel complexer en fluctueerde ook sterk over de tijd. Zo was het landelijke advies in het begin van onze onderzoeksperiode nog om kinderen niet te laten testen, maar mocht dit later wel. Ook het grootschalig testen in verpleeg- en verzorgingshuizen en onder zorgpersoneel door andere partijen dan de GGD was een landelijk advies. Daarnaast werden op basis van lokaal beleid bepaalde wijken of gemeenten om verschillende redenen actief benaderd voor testen door de GGD, zoals een veronderstelde verminderde testbereidheid of een lokale uitbraak. De GGD heeft echter geen zicht op het testbeleid van alle individuele werkgevers in de onderzochte regio’s. Daardoor is het lastig inschatten hoe groot het effect daarvan op de datakwaliteit van specifieke groepen werknemers geweest is.

De datakwaliteit van een populatiegroep is dus goed wanneer binnen deze groep veel mensen door de GGD getest mochten worden en daartoe ook bereid waren. In onze casus werd de GGD-teststraat vooral bezocht door inwoners uit huishoudens met kinderen, met een hoog tot zeer hoog huishoudinkomen en loon (inclusief dat van directeur-grootaandeelhouders) als belangrijkste bron van dat huishoudinkomen.

Wanneer veel mensen binnen een populatiegroep niet door de GGD, maar wel elders getest mochten worden, is de registratiekwaliteit van positieve uitslagen goed, maar die van negatieve uitslagen slecht. Populatiegroepen met veel ontbrekende negatieve testuitslagen in de data zijn te identificeren op basis van het aandeel positieven dat buiten de teststraat van de GGD werd vastgesteld. In onze casus zagen we bijvoorbeeld dat dit aandeel hoog was onder inwoners van 65 jaar en ouder, bewoners van instellingen en tehuizen, en inwoners van wie het pensioen de belangrijkste bron van het huishoudinkomen was.

Voor populatiegroepen die helemaal nergens getest mochten of wilden worden is de datakwaliteit zeer slecht. Deze populatiegroepen zijn op basis van de beschikbare data lastig te identificeren. Op deze manier heeft de interactie tussen testbeleid, testbereidheid en registratiebeleid dus geleid tot populatieverschillen in datakwaliteit.

### Potentieel effect van registratie- en testbeleid op de berekende besmettingsgraad

**Figure 2:**
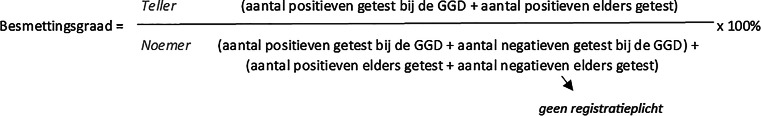

Populatieverschillen in datakwaliteit leiden vervolgens tot populatieverschillen in betrouwbaarheid van de berekende besmettingsgraad. De besmettingsgraad kan worden berekend op basis van de formule in fig. 2.

Voor de populatiegroepen met een goede datakwaliteit is de besmettingsgraad berekend op basis van de beschikbare registratiedata vrij betrouwbaar. Voor populatiegroepen met een goede datakwaliteit van positieven, maar een slechtere datakwaliteit van negatieven zal de berekende besmettingsgraad altijd een overschatting van de werkelijkheid zijn. Het werkelijke aantal negatieven, inclusief het aantal vastgesteld buiten de GGD-teststraat dat niet werd geregistreerd, kan immers alleen maar hoger zijn dan het geregistreerde aantal negatieven. Voor populatiegroepen met een zeer slechte datakwaliteit is de besmettingsgraad niet betrouwbaar te berekenen. Populatiegroepen waarvan de betrouwbaarheid van de besmettingsgraad niet even groot is, kunnen vanzelfsprekend niet eerlijk worden vergeleken. In onze casus konden daardoor bijvoorbeeld verschillen in besmettingsgraad tussen werknemers uit verschillende arbeidssectoren of tussen inwoners uit particuliere huishoudens en (zorg)instellingen niet betrouwbaar worden geanalyseerd.

### Evaluatie: gevolgen voor datagestuurd werken in de publieke gezondheidssector

Deze beperkingen impliceren echter niet dat datagestuurd werken helemaal niet mogelijk is, maar onderbouwen wel het belang van vergelijkbare datakwaliteit. De verschillen in besmettingsgraad tussen uiteenlopende typen huishoudens binnen zorginstellingen kunnen bijvoorbeeld wel worden onderzocht. Voor beide typen huishoudens was immers hetzelfde test- en registratiebeleid van kracht, en reflecteert de berekende besmettingsgraad een onderschatting van de werkelijkheid. Door eerlijke vergelijkingen te maken, wordt betrouwbaardere sturingsinformatie verzameld. Dit voorkomt verspreiding van misinformatie en kan daarmee uiteindelijk bijdragen aan een verminderde stigmatisering van populatiegroepen. Sec sturen op geografische statistieken, zoals de besmettingsgraad op wijk- en buurtniveau, is vanwege de beschreven vertekening door test- en registratiebeleid af te raden. Binnen, maar ook tussen de wijken kan de datakwaliteit immers sterk variëren.

## Beschouwing en aanbevelingen voor de toekomst

Deze casus heeft ons een aantal waardevolle inzichten opgeleverd. Bestaande wettelijke kaders, procedures, taakomschrijvingen en registratiesystemen die goed werkten in de reguliere praktijk van infectieziektebestrijding door GGD’en bleken niet altijd voldoende in een crisissituatie met een aard en omvang als die van de COVID-19-pandemie. Om onderzoekers van GGD’en in de toekomst beter te faciliteren in het creëren van betrouwbare sturingsinformatie zijn twee belangrijke aanbevelingen geformuleerd.

### Aanbeveling 1: investeer in één betrouwbaar registratiesysteem inclusief negatieve testuitslagen

We adviseren te investeren in een eenduidig betrouwbaar registratiesysteem van positieve én negatieve testuitslagen voor alle mogelijke testaanbieders en voor alle mogelijke virusinfecties. Op deze manier wordt een groot deel van de in onze casus geïllustreerde populatieverschillen in datakwaliteit voorkomen, omdat voor alle populatiegroepen evenveel zicht is op de negatieve testuitslagen. Naast betrouwbaardere schattingen van populatieverschillen in de besmettingsgraad zou een dergelijke registratie ook waardevollere sturingsinformatie bieden over testgedrag. Dan kan per populatiegroep in kaart gebracht worden welk percentage zich heeft laten testen, in plaats van welk percentage zich heeft laten testen bij de teststraat van de GGD.

### Aanbeveling 2: bouw aan vertrouwen en geef inwoners inspraak

Testbereidheid is in onze casus een belangrijke determinant van datakwaliteit, maar dit is lastig meetbaar te maken. Idealiter zouden we in toekomstige gezondheidscrises niet alleen betrouwbaardere maar ook relevantere sturingsinformatie willen verzamelen. Dat wil zeggen, informatie waar een beleidsmedewerker of bestuurder direct interventies op kan ontwikkelen, zoals de onderliggende oorzaken van een verminderde testbereidheid. We weten bijvoorbeeld dat wantrouwen in de overheid en onzekerheid over privacy redenen kunnen zijn voor verminderde testbereidheid. Ons tweede advies is dan om ook te werken aan het vertrouwen van inwoners door hen inspraak te geven in de ontwikkeling van publiek gezondheidsbeleid. Idealiter wordt samen met hen een systeem ontwikkeld waarin ze altijd inzage hebben in hun eigen publieke gezondheidsdata, waaraan ze actief informatie kunnen toevoegen en waarbij ze bij aanvang van een toekomstige crisis direct zelf kunnen aangeven of en onder welke voorwaarden hun data gebruikt mogen worden voor onderzoek dat is gericht op het verkleinen van gezondheidsverschillen.

## References

[1] ZonMw. Populatieverschillen in gediagnosticeerde covid-19 prevalentie in Nederland: Welke groepen lopen het hoogste risico? 2021. https://www.zonmw.nl/nl/over-zonmw/coronavirus/programmas/project-detail/covid-19-programma/sociaaleconomische-verschillen-in-gediagnosticeerde-covid-19-prevalentie-in-zuid-west-nederland/resultaten/. Geraadpleegd op: 30 nov 2021.

[2] CBS. Catalogus microdata. Den Haag: Centraal Bureau voor de Statistiek. https://www.cbs.nl/nl-nl/onze-diensten/maatwerk-en-microdata/microdata-zelf-onderzoek-doen/catalogus-microdata. Geraadpleegd op: 4 jul 2021.

[3] Mackenbach J (2000). Dwalingen in de methodologie. XXVI. De ecologische valkuil en zijn minder bekende tegenhanger, de atomistische valkuil. Ned Tijdschr Geneeskd.

